# Aesthetic and Incentive Salience of Cute Infant Faces: Studies of Observer Sex, Oral Contraception and Menstrual Cycle

**DOI:** 10.1371/journal.pone.0065844

**Published:** 2013-05-29

**Authors:** Reiner Sprengelmeyer, Jennifer Lewis, Amanda Hahn, David I. Perrett

**Affiliations:** University of St. Andrews, School of Psychology and Neuroscience, St. Andrews, Scotland; University of Bologna, Italy

## Abstract

Infant cuteness can influence adult-infant interaction and has been shown to activate reward centres in the brain. In a previous study, we found men and women to be differentially sensitive to small differences in infant facial cuteness, with reproductive hormone status as the potential underlying cause. It is unclear, however, whether reproductive hormone status impacts on the aesthetic and incentive salience of infant faces.

To address this question, we conducted two interlinked studies. We used static images of the same smiling and neutral-looking infant faces in both a rating task, in which participants had to rate the cuteness of infant faces (aesthetic salience - ‘liking’), and a key-press task, in which participants could prolong or shorten viewing time of infant faces by rapid alternating key-presses (incentive salience - ‘wanting’).

In a first study, we compared the performance of men, women who are taking oral contraceptives, and regularly cycling women. In this study, we found a significant correlation between cuteness ratings within and between groups, which implies that participants had the same concept of cuteness. Cuteness ratings and effort to look at faces was linked regardless of sex and reproductive hormone status, in that cute faces were looked at for longer than less cute faces. A happy facial expression contributed only marginally to the incentive salience of the face.

To explore the potential impact of reproductive hormone status in more detail, we followed a subset of regularly cycling women during the menstrual, follicular and luteal phases of their cycle. The aesthetic and incentive salience of infant faces did not change across the menstrual cycle.

Our findings suggest that reproductive hormone status does not modulate the aesthetic and incentive value of infant faces.

## Introduction

The ethologist Konrad Lorenz put forward the idea of the *Kindchenschema* as an innate neuro-cognitive releasing mechanism, which elicits a positive orientation towards an infant as well as care-giving behaviour [1]. This mechanism is triggered by neotonous features, amongst these a large forehead, chubby cheeks, and big round eyes below or near the horizontal midline of the skull. Infants with these features are described as being cute. The claim that cuteness can elicit positive responses has been confirmed subsequently by a number of studies. Cute infants are rated as more intelligent [2], [3], are looked at for longer [4], [5], elicit a stronger care-giving desire [6] and enjoy more ‘affectionate’ interactions with their mothers [7].

The *Kindchenschema* is conceptualised as a biological mechanism, hence in a previous study we investigated the potential role of reproductive hormone status in cuteness processing. We used computer-manipulated images of real infants to investigate the ability to see small differences in cuteness between infant faces. We found that young women aged between 19 and 51 years were more sensitive to differences in infant cuteness than men, regardless of age, and older women aged 53 to 60 years. Follow-up studies further showed that pre-menopausal women and young women taking oral contraceptives (which raise hormone levels artificially) were more sensitive to small differences in cuteness than their respective comparison groups [8]. Lobmaier and co-workers reported that women are more sensitive to infant cuteness than men, while men and women do not differ in their ability to extract information about emotion or age from infant faces [9].

Although our previous studies have established a potential link between reproductive hormone status and cuteness sensitivity, we currently do not know whether reproductive hormone status modulates the aesthetic salience (‘liking’) or the incentive salience (‘wanting’) of infant faces, and whether aesthetic salience and incentive salience of infant faces are linked. Both ‘liking’ (as assessed with a rating task) and ‘wanting’ (as assessed with a ‘pay per view task’) are dissociable components of the reward process and associated with separable neural structures; ‘liking’ is associated with fronto-temporal brain regions, whereas ‘wanting’ is associated with mesolimbic brain regions [10], [11], [12].

Four studies so far have addressed the question whether or not ‘liking’ or ‘wanting’ of infant faces differ between men and women. Two studies looked at the responses of men and women to infant faces with and without facial abnormalities [13], [14], one study looked at cute and less cute infant faces [15], and one study used computer-manipulated cute and less cute infant faces as well as attractive and less attractive adult faces [16]. In three of the four studies, women rated non-disfigured infant faces as more attractive than did men (‘liking’), but in a ‘pay per view’ task, both men and women showed the same effort to look at these faces (‘wanting’) [13], [14], [15]. In addition, cute infant faces were ‘liked’ and ‘wanted’ more by men and women than less cute infant faces [15]. Contrasting findings came from Hahn and co-workers [16], who showed that women exerted the same amount of effort to look at cute infant faces and attractive opposite sex faces, while men tried to shorten viewing time for infant faces when they had the opportunity to look at attractive female faces. The two studies looking at aesthetic and incentive salience of disfigured infant faces showed discrepant results. In one study [14], women gave higher ratings then men, but viewing time for faces with abnormalities did not differ between men and women. In the second study [13], men and women gave similar ratings for infant faces with abnormalities, but viewing time was longer for men than for women. This difference may well be caused by the kind of stimuli; one study used infant faces with cleft lip only [14], the other study used infant faces with a variety of facial abnormalities [13].

None of the authors of these four studies took reproductive hormone status as a potential modulator of performance into consideration.

To explore the role of reproductive hormone status on aesthetic salience and incentive salience of infant faces, we first looked at men, and women taking and not taking oral contraceptives, and then in a second study, we looked at regularly cycling women while in the menstrual, follicular and luteal phases of their cycle. We used an established ‘pay per view’ key-press paradigm [10], in which participants viewed a series of individual infant faces on a screen, which changed automatically after a few seconds. Participants could increase viewing time for faces they liked to look at for longer, and decrease viewing time for faces they did not want to see that long by rapidly pressing alternate buttons on the keyboard. We used viewing time as a quantitative measure of motivation strength to orient towards infants (‘wanting’). In addition we asked participants to rate the cuteness of the infants used in the key-press task (‘liking’). We also explored the effect of positive emotions expressed by infants on ‘liking’ and ‘wanting’, using neutral-looking and smiling infant faces.

## Methods

The study was approved by the Ethics Committee of St Andrews University. All participants gave informed written consent to take part in the study.

### Participants - Cross sectional study

Twenty-three regularly cycling women (mean age  = 20.8 years, SD 1.9), 25 women taking oral contraceptives (mean age  = 19.6 years, SD 1.4), and 26 men (mean age  = 20.6 years, SD 2.8) took part in the study. The groups did not differ in respect to age (one-way ANOVA, F = 1.93, df 2,70 ; p = .15). None of the participants had own children. All participants taking oral contraceptives took the monophasic combined pill (containing oestrogen and progesterone) and had been taking the pharmacologically active hormonal pill for at least 2 days prior to testing (range 2–21 days). Half of these women used Microgynon30 (Bayer) with 35 µg ethinylestradiol and 150 µg levonorgestrel, while the other half used oral contraceptives from other manufacturers. These contraceptive pills included either 20 or 35 µg ethinylestradiol, and varying progesterone components (e.g. 100–150 µg levonorgestrel, 3000 µg drospirenone, 250 µg norgestimate, 75 µg gestodene, or 1000 µg norethindrone acetate). Overall, this specific distribution of medication is typical and does not differ from the spread of medication found in previous studies on student populations in Scotland [8].

(N.B. The cross-sectional study reports only results from participants’ first testing which took place in any one of the menstrual, follicular or luteal phases.)

### Participants - Across menstrual cycle study

From the group of regularly cycling women, 11 participants (mean age  = 20.5 years, SD 1.4) were tested during in the menstrual, follicular (i.e. ovulation) and luteal phases of their cycle. Testing in the menstrual phase took place between days 1 and 5 of the cycle, as confirmed by onset of menstruation. Ovulation was determined by tests, which detect the surge in luteinising hormone (LH) that takes place immediately preceding ovulation. Participants performed the experimental tasks no later than 72 hours after having a positive LH-test result. This typically occurred between days 12 and 16 of the cycle. The luteal session was scheduled between days 19 and 28 of the cycle, based on the last menstruation and results from the ovulation prediction kits. The women were recruited at different phases of their menstrual cycle in order to counterbalance the order of tests.

### Stimuli

A total of 56 images depicting faces of infants between 6 and 12 months of age were used as stimuli for both the key-press and the rating task. Half of the faces showed a happy facial expression (smiling or laughing, happy set), the other half displayed a neutral facial expression (neutral set). The background of the pictures was masked to standardise image presentation. The selection process of the stimuli involved one rater, who explicitly looked for and selected 28 faces with happy facial expressions and 28 neutral-looking faces. It should be noted that this initial selection does not form a continuum of faces ranging from neutral to happy, but is bimodal by design. To further validate the 2 sets *post-hoc*, we asked 7 raters (3 male, 4 female), with a mean age of 28.4 years (SD 4.4), to decide whether the 56 faces used in the study displayed either a happy or neutral facial expression. Responses were given a score of 1 if a face was seen as happy, and a score of 0 if the face was seen as neutral. For each face, we calculated the average score, which should be near 0 for the neutral faces, and near 1 for the happy faces. The 28 faces from the neutral set had an overall mean score of 0.16 (SD 0.11), the 28 faces from the happy set an overall mean score of 0.97 (SD 0.03). The mean scores of the two sets differed significantly (Wilcoxon Test (one-tailed): z = –2.37; p<0.01, effect size: r = –0.64). The results validate the separation of the stimuli into a happy and a neutral set.

### Rating task (‘Liking’)

Each of the 56 images was presented individually and without a time limit, in random order on a computer screen. Underneath each picture was a Likert scale ranging from 1 (‘Not very cute’) to 5 (‘Very cute’). Participants had to rate the cuteness of each individual face by using the keys 1 to 5 on the computer’s keyboard. The next image appeared following each decision.

### Key-press task (‘Wanting’)

Images were presented individually in random order on a computer screen, changing every 4 seconds if no further action was taken. Participants were able to increase or decrease the viewing time of each image by exerting effort through key-pressing. The maximum length of time the image could be viewed was 16 seconds, the minimum length of time was 2 seconds. To visualise the amount of time available for viewing the individual infant face, a vertical green bar was situated to the left of the image, which decreased in length as the amount of viewing time left decreased. Pressing the N and M keys added length to the green bar and prolonged viewing time, pressing the Z and X keys shortened the length of the bar and reduced the viewing time. The dependent variable was viewing time.

## Results

### Cross-sectional study

To ensure that cuteness ratings were consistent before combining the data sets for further analysis, we first tested correlations of cuteness ratings within and between groups. We calculated Cronbach’s α for each group as a measure of internal consistency, and, in a further step, correlated ratings of one group with the ratings of all other groups. Cronbach’s α for women taking oral contraceptives and for regularly cycling women was.92. Cronbach’s α for men was.95. Cuteness ratings from men and from women taking oral contraceptives were significantly correlated (r = .85, p<.001), as were the ratings from men and from regularly cycling women (r = .87, p<.001). Cuteness ratings from women taking oral contraceptives and from regularly cycling women correlated significantly (r = .75, p<.001).

**Figure 1:**
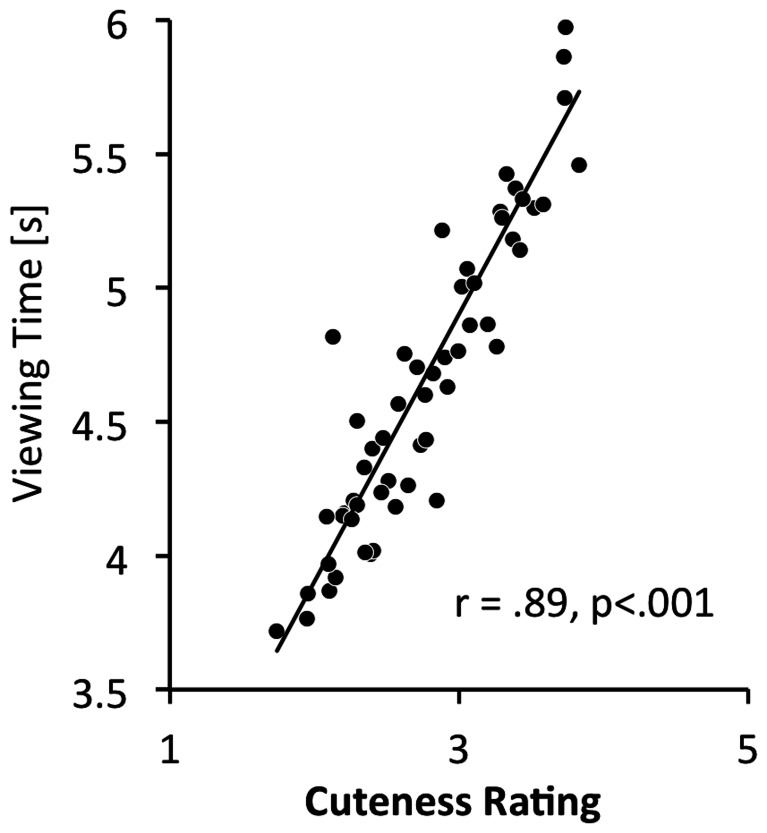
Correlation between overall cuteness rating and viewing times.

Viewing time and cuteness rating were significantly correlated with r = .89 (p<.001). See Figure 1 for details.

To explore the impact of cuteness on ‘liking’ and ‘wanting’ independently, we calculated for each participant the average rating of the 1^st^ quartile (the 25% of faces with lowest cuteness ratings), the average of the 2^nd^ and 3^rd^ quartiles, (faces with intermediate cuteness ratings), and the average of the 4^th^ quartile (faces rated high on cuteness), separately for the happy and neutral faces. The resulting variables were ‘Rating – low’, ‘middle’, and ‘high’. Since 56, the total number of happy and neutral -looking faces, cannot be divided by three, we decided to calculate the variables ‘Rating – low’, ‘middle’, and ‘high’ as described above in order not to lose information by discarding items.

The same sets of pictures were also used to calculate the corresponding variables ‘Viewing time – low’, ‘middle’, and ‘high’ for viewing time on the key-press task for the happy and neutral faces.

#### Rating task (‘liking’)

A non-parametric comparison of the overall cuteness rating score (for details see Table 1, row 9) showed no significant group differences (Kruskal-Wallis ANOVA χ^2^ (df 2) 0.21, p = 0.90). Three *post-hoc* Mann-Whitney Tests were performed (men vs. regularly cycling women, men vs. women taking oral contraceptives, regularly cycling women vs. women taking oral contraceptives) to calculate effect sizes (–0.40≤z≤–0.08; 0.69≤p≤0.94, effect size: –0.05≤r≤–0.01).

**Table 1:** Cuteness ratings for women taking oral contraceptives, regularly cycling women, and men.

		Women (Pill)		Women (cycling)		Men		
		Mean	(SD)	Mean	(SD)	Mean	(SD)	sign.
**1 Neutral Low**		1.9	(0.5)	2.1	(0.6)	2.1	(0.5)	n.s.
**2 Neutral Middle**		2.7	(0.5)	2.7	(0.5)	2.7	(0.6)	n.s.
**3 Neutral High**		3.5	(0.7)	3.3	(0.7)	3.3	(0.6)	n.s.
**4 Neutral Overall**		2.7	(0.5)	2.7	(0.5)	2.7	(0.5)	n.s.
**5 Happy Low**		2.1	(0.6)	2.2	(0.8)	2.2	(0.7)	n.s.
**6 Happy Middle**		2.8	(0.5)	2.7	(0.7)	2.7	(0.6)	n.s.
**7 Happy High**		3.7	(0.7)	3.6	(0.7)	3.4	(0.6)	n.s.
**8 Happy Overall**		2.9	(0.5)	2.9	(0.6)	2.8	(0.6)	n.s.
**9 Overall**		2.8	(0.4)	2.8	(0.5)	2.7	(0.4)	n.s.

We also looked at the overall rating of happy and neutral infant expressions separately (for details see Table 1, rows 4 and 8, respectively). There was no significant difference between groups for happy facial expressions (Kruskal-Wallis ANOVA χ^2^ (df 2) 0.79, p = 0.67; Mann-Whitney Tests: –1.07≤z≤–0.15; 0.28≤p≤0.88, effect size: –0.15≤r≤–0.02), or neutral expressions (Kruskal-Wallis ANOVS χ^2^ (df 2) 0.10, p = 0.95; Mann-Whitney Tests: –0.30≤z≤–0.09; 0.80≤p≤0.93, effect size: –0.04≤r≤–0.01).

We analysed the performance of the three groups for the different levels of cuteness (low, middle and high) for happy and neutral facial expressions separately.

We found no significant differences for faces with happy expressions with low (Kruskal-Wallis ANOVA χ^2^ (df 2) 0.31, p = 0.86; Mann-Whitney Tests: –0.50≤z≤–0.02; 0.62≤p≤0.98, effect size: –0.07≤r≤–0.0), middle (Kruskal-Wallis ANOVA χ^2^ (df 2) 1.22, p = 0.55; Mann-Whitney Tests: –1.31≤z≤–0.26; 0.19≤p≤0.80, effect size: –0.18≤r≤–0.04), and high cuteness ratings (Kruskal-Wallis ANOVA χ^2^ (df 2) 2.49, p = 0.29; Mann-Whitney Tests: –1.56≤z≤–0.05; 0.12≤p≤0.73, effect size: –0.22≤r≤–0.05). See Table 1, rows 5 to 7 for details.

The analysis of neutral facial expressions revealed similar results. There were no significant group differences for faces with low (Kruskal-Wallis ANOVA Test χ^2^ (df 2) 0.94, p = 0.63; Mann-Whitney Tests: –0.92≤z≤–0.33; 0.36≤p≤0.74, effect size: –0.13≤r≤–0.05), middle (Kruskal-Wallis ANOVA χ^2^ (df 2) 0.22, p = 0.90; Mann-Whitney Tests: –0.47≤z≤0.08; 0.64≤p≤0.93, effect size: –0.07≤r≤–0.01), and high cuteness ratings (Kruskal-Wallis ANOVA χ^2^ (df 2) 1.55, p = 0.46; Mann-Whitney Tests: –1.23≤z≤–0.46; 0.22≤p≤0.64, effect size: –0.17≤r≤–0.07). See Table 1, rows 1 to 3 for details.

We also tested for significant within-group differences in cuteness ratings of happy and neutral expressions (for details see Table 1, Neutral Overall vs. Happy Overall). There was no significant difference in men (Wilcoxon Test: z = –0.37; p = 0.71, effect size: r = –0.05), regularly cycling women (Wilcoxon Test: z = –0.53; p = 0.60, effect size: r = –0.08), and women taking oral contraception (Wilcoxon Test: z = –1.36; p = 0.17, effect size: r = –0.20).

#### Key-press task (‘wanting’)

**Figure 2:**
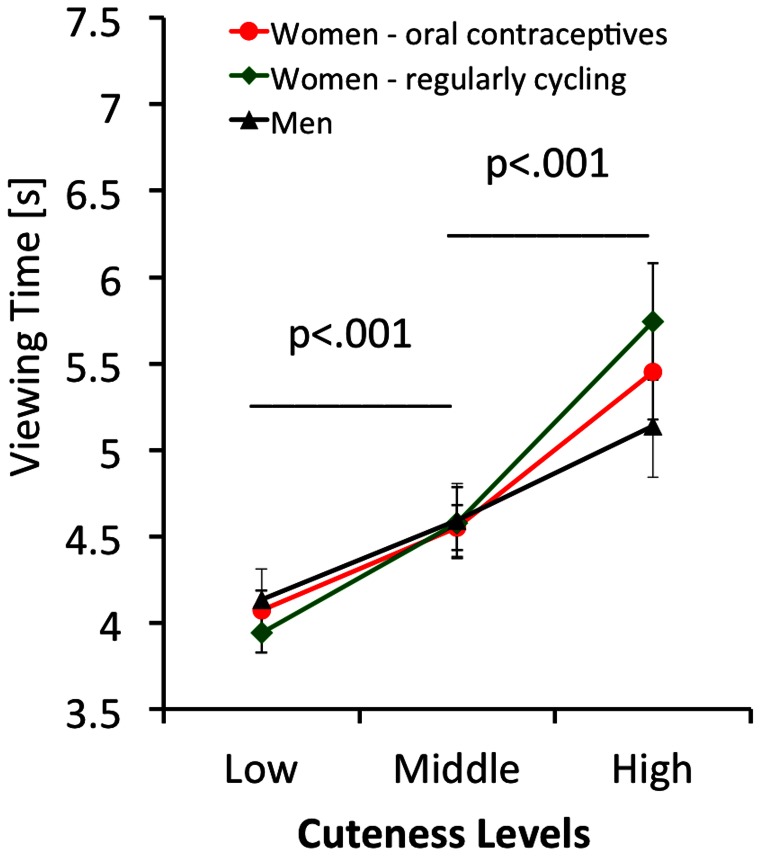
Viewing times of men, women taking oral contraceptives, and regularly cycling women for faces rated as low, medium and high in cuteness. Error bars give mean +/– SE.

**Figure 3:**
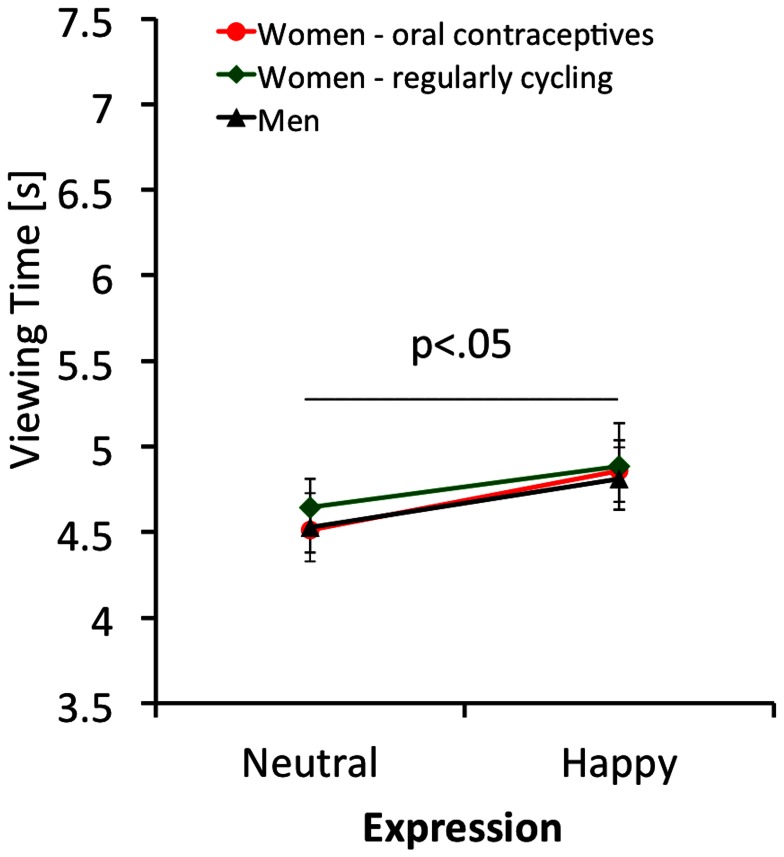
Viewing times of men, women taking oral contraceptives, and regularly cycling women for faces displaying neutral and happy expressions. Error bars give mean +/– SE.

To determine whether motivation to look at infants is modulated by sex and reproductive hormone status, we performed a mixed between/within ANOVA (with level of cuteness and expression as within-subject factors, and groups as a between subject factor) using the viewing time data from the key-press task as the dependent variable. Kolmogorov-Smirnov tests established normal distribution of the viewing time data with probabilities ranging between p = .21 and p = .99 for all measures. Since sphericity cannot be assumed, we performed Greenhouse Geisser corrections. There was a significant effect of cuteness (F = 66.35; df 1.346,95.559; p<.001; η^2^ = .480), in that cuter infants were looked at for longer (see Figure 2). There was also a significant effect of expression (F = 4.30; df 1,71; p<.05; η^2^ = .057), in that happy faces were viewed for longer than neutral faces (see Figure 3). We did not find a significant groups effect (F = 0.13; df 1,71; p = .88; η^2^ = .004), and all 2-way interactions failed to reach significance. The expression x group interaction was not significant (F = 0.26; df 2,71; p = .77; η^2^ = .007) as was the cuteness x group interaction (F = 1.96; df 2.692,95.559; p = .13; η^2^ = .052) and the expression x cuteness interaction (F = 1.86; df 1.657,117.637; p = .17; η^2^ = .026). The 3-way interaction also failed to reach significance (F = 0.45; df 3.314,117.637; p = .74; η^2^ = .012).

### Across menstrual cycle study

The relative levels of oestrogen and progesterone vary across the menstrual cycle, in that (a) levels of oestrogen and progesterone are relatively low during the menstrual phase, (b) the level of oestrogen is high in the late follicular phase relative to the level of progesterone, (c) the level of progesterone is high relative to the level of oestrogen during the luteal phase. In this study, we wanted to explore the potential impact of menstrual cycle phase on cuteness rating and viewing time.

#### Rating task (‘liking’)

A non-parametric comparison of the overall rating score (for details see Table 2, row 9) across the follicular, luteal and menstrual phase of the cycle showed no significant differences (Friedman Test χ^2^ (df 2,11) 2.36, p = 0.31). Three *post-hoc* Wilcoxon Tests were performed (menstrual phase vs. follicular phase, follicular phase vs. luteal phase, menstrual phase vs. luteal phase) to calculate effect sizes (–0.62≤z≤–0.27; 0.53≤p≤0.79, effect size: –0.57≤r≤–0.06).

**Table 2:** Cuteness ratings for the different phases of the menstrual cycle.

		Menstrual		Follicular		Luteal		
		Mean	SD	Mean	SD	Mean	SD	sign.
**1 Neutral Low**		2.4	0.7	2.2	0.9	2.3	0.6	n.s.
**2 Neutral Middle**		2.8	0.5	2.8	0.7	2.8	0.4	n.s.
**3 Neutral High**		3.5	0.8	3.7	0.5	3.7	0.8	n.s.
**4 Neutral Overall**		2.9	0.5	2.9	0.6	2.9	0.5	n.s.
**5 Happy Low**		2.4	0.8	2.3	1	2.5	0.9	n.s.
**6 Happy Middle**		2.9	0.8	2.8	1	3	0.7	n.s.
**7 Happy High**		3.6	0.8	3.5	0.8	3.8	0.4	n.s.
**8 Happy Overall**		3	0.7	2.9	0.9	3.1	0.6	n.s.
**9 Overall**		2.9	0.5	2.9	0.7	3	0.5	n.s.

We also looked at the overall rating of happy and neutral infant expressions separately (for details see Table 2, rows 4 and 8, respectively). There was no significant difference between cycle stages for either happy facial expressions (Friedman Test χ^2^ (df 2,11) 0.73, p = 0.70; Wilcoxon Tests: –0.66≤z≤–0.53; 0.50≤p≤0.59, effect size: –0.14≤r≤–0.11) or neutral expressions (Friedman Test χ^2^ (df 2,11) 0.55, p = 0.76; Wilcoxon Tests: −0.53≤z≤−0.0; 0.59≤p≤1.0, effect size: −0.57≤r≤−0.0).

In a further step, we analysed the performance across cycle stages for the different levels of cuteness (low, middle and high) for happy and neutral facial expressions separately.

For happy facial expressions, we found no significant differences for faces with low (Friedman Test χ^2^ (df 2,11) 0.35, p = 0.84; Wilcoxon Tests: −0.71≤z≤−0.46; 0.48≤p≤0.65, effect size: −0.15≤r≤−0.10), middle (Friedman Test χ^2^ (df 2,11) 0.91, p = 0.64; Wilcoxon Tests: −0.62≤z≤−0.31; 0.53≤p≤0.76, effect size: −0.11≤r≤−0.07), and high cuteness ratings (Friedman Test χ^2^ (df 2,11) 0.63, p = 0.73; Wilcoxon Tests: −1.03≤z≤−0.35; 0.30≤p≤0.73, effect size: −0.22≤r≤−0.07). See Table 2, rows 5 to 7 for details.

We found similar results for neutral facial expressions. There were no significant differences between cycle stages for faces with low (Friedman Test χ^2^ (df 2,11) 1.76, p = 0.41; Wilcoxon Tests: −0.84≤z≤−0.51; 0.40≤p≤0.61, effect size: −0.18≤r≤−0.11), middle (Friedman Test χ^2^ (df 2,11) 0.18, p = 0.93; Wilcoxon Tests: −0.80≤z≤0.0; 0.42≤p≤1.0, effect size: −0.17≤r≤−0.0), and high cuteness ratings (Friedman Test χ^2^ (df 2,11) 0.63, p = 0.72; Wilcoxon Tests: −1.26≤z≤−0.09; 0.21≤p≤0.93, effect size: −0.27≤r≤−0.02). See Table 2, rows 1 to 3 for details.

We also tested for potential significant differences in ratings between happy and neutral expressions for the three cycle stages separately. There was no significant difference for the menstrual (Wilcoxon Test: z = −0.27; p =  0.79, effect size: r = −0.06), follicular (Wilcoxon Test: z = −0.18; p =  0.86, effect size: r = −0.04), and luteal phase of the menstrual cycle (Wilcoxon Test: z = −0.53; p =  0.59, effect size: r = −0.11). For details see Table 2, Neutral Overall vs. Happy Overall.

#### Key-press task (‘wanting’)

**Figure 4:**
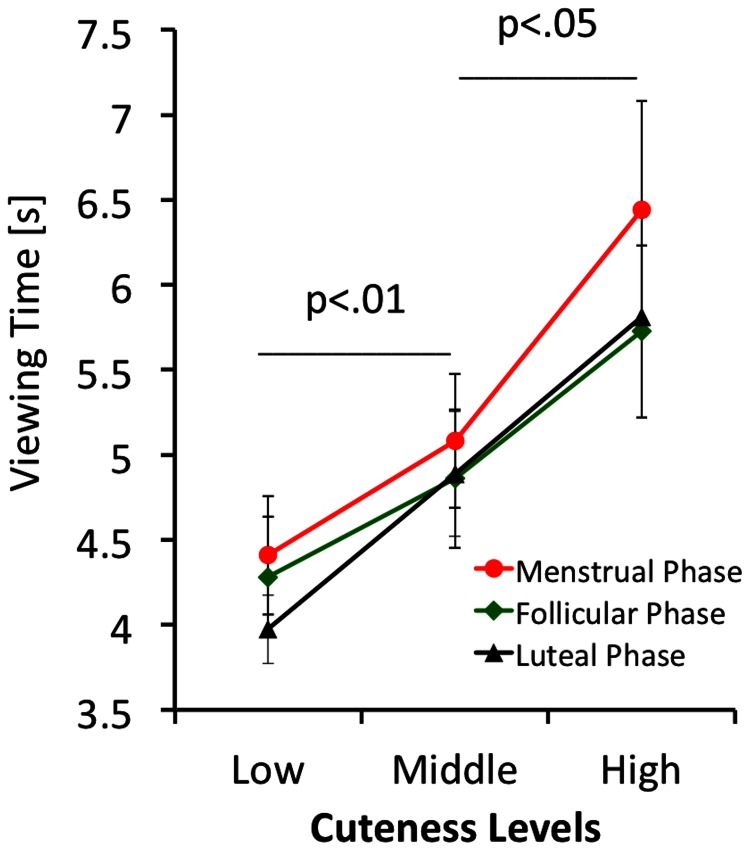
Viewing times of regularly cycling women for the menstrual, follicular and luteal phases of the cycle for faces rated as low, medium and high in cuteness. Error bars give mean +/− SE.

**Figure 5:**
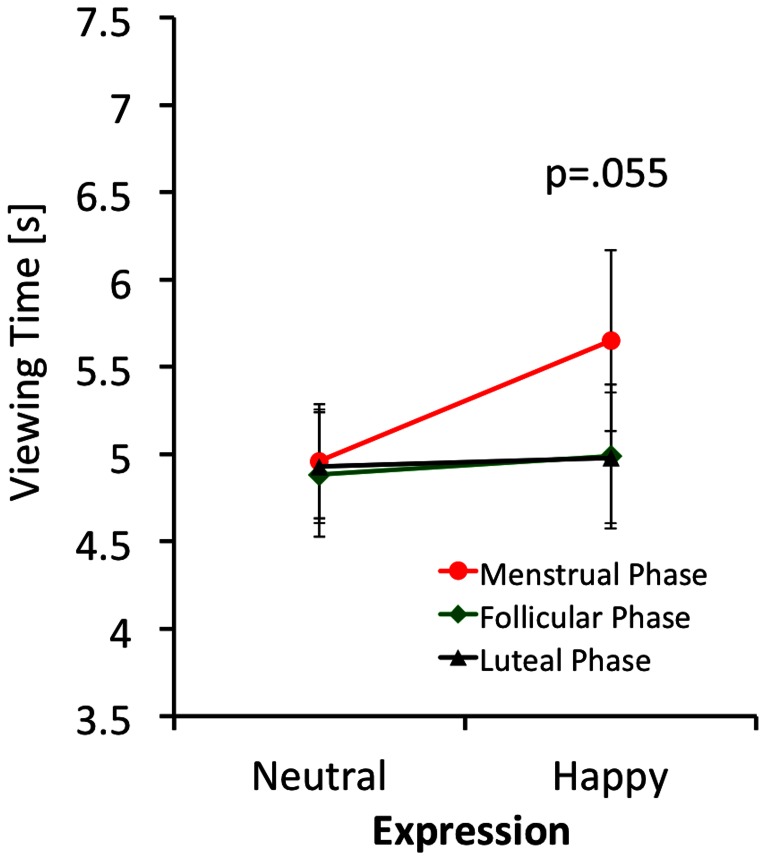
Viewing times of regularly cycling women for the menstrual, follicular and luteal phases of the cycle for faces displaying neutral and happy facial expressions. Error bars give mean +/− SE.

A mixed between/within ANOVA was performed (with cycle phase, level of cuteness, and expression as within-group factors) using viewing time as the dependent variable. Kolmogorov-Smirnov tests established normal distribution of the viewing time data with probabilities ranging between p = .19 and p = .89 for all measures. Since sphericity cannot be assumed, we performed Greenhouse Geisser corrections. There was a significant effect of cuteness (F = 12.21; df 1.270,12.701; p<.01; η^2^ = .550) (see Figure 4 for details), no significant effect of expression (F = 0.15; df 1,10; p = .71; η^2^ = .015) or menstrual cycle phase (F = 1.30; df 1.819, 18.185; p = .30; η^2^ = .115), but a trend towards significance for the expression x menstrual cycle phase interaction (F = 3.78; df 1.560, 15.60; p = .055; η^2^ = .274). As can be seen in Figure 5, happy faces were looked at for longer in the menstrual phase of the cycle. All other 2-way interactions, and the 3-way interaction were non-significant, suggesting that hormonal fluctuations during the menstrual cycle do not modulate ‘wanting’ of infant faces. The cuteness x cycle phase interaction was not significant (F = 0.88; df 1.983, 19.835; p = .43; η^2^ = .081) as was the cuteness x expression interaction (F = 2.36; df 1.385, 13.852; p = .14; η^2^ = .191) and the cycle phase x cuteness x expression interaction (F = 0.52; df 2.635, 26.352; p = .65; η^2^ = .050).

## Discussion

Following up the idea of involvement of reproductive hormone status in cuteness processing [8], we investigated whether reproductive hormone status modulates the aesthetic (‘liking’) and incentive salience (‘wanting’) of happy and neutral infant faces.

We used two established tasks [10], a rating task, in which participants had to judge the cuteness of infant faces (‘liking’), and a key-press task, in which participants could prolong or shorten viewing time of happy and neutral-looking infant faces by rapid alternating key-presses (‘wanting’).

In our cross-sectional study, we first looked at the cuteness rating of men, regularly cycling women, and women taking oral contraceptives.

Correlational statistics revealed that the internal consistency within each group was high as well as the correlation of cuteness ratings between groups. This suggests that the concept of cuteness applied to the individual infant faces in the rating task was similar for all participants and probably not modulated by reproductive hormone level.

In accordance with this, planned non-parametric comparisons revealed no significant differences in overall cuteness ratings between groups. Effect sizes for these comparisons were very small. We also compared the cuteness ratings of the three groups for neutral and happy facial expressions, as well as for the three levels of cuteness for both the neutral and happy expressions. All between-group comparisons gave non-significant results with small to very small effect sizes.

This finding is in contrast to other studies [13], [14], [15], which reported that women gave higher attractiveness ratings for infant faces than men. The reason for this discrepancy remains unclear. It may be due to social norms (which are expressed more prominently in the other samples) in that women are expected to be more interested in infants; to show this, they may rate infants more favourably than men. Alternatively, the (predominantly young) male participants may find it ‘uncool’ to like babies. A different reason for the discrepant results might be related to the way the ratings were performed. Parsons and co-workers and Yamamoto and co-workers [13], [14], [15] used visual analogue scales, which are potentially more sensitive, while we used a 5-point Likert scale. This methodologically important aspect needs to be explored further.

Previous research also showed that happy infant faces were more liked than neutral ones [2], [3]. To explore this, we compared cuteness ratings of pictures of infants with happy and neutral facial expressions. We found no significant differences in cuteness ratings between happy and neutral facial expressions within each of the three groups. For these comparisons, the effect sizes for men and women not taking oral contraception were very small, the effect size for women taking oral contraception was small to medium. Given that faces had to be rated in respect to cuteness, these results imply that the concept of cuteness is distinct from positive emotions expressed in a face.

For the key-press task, we first looked at the correlation of average rating and average viewing time across participants for each face. The correlation was high, in that the cuter a face, the stronger its incentive value. This correlational finding is mirrored in the highly significant cuteness effect of the ANOVA of the key-press task. The effect size for this was large. There was also a significant effect of emotion, but the effect size for this was rather small (see Figure 3 for illustration). Overall, the incentive salience of infant faces seems to be modulated by infant facial cuteness and to a lesser extent by a positive facial expression (as implied by the emotion effect). A non-significant group effect for a key-press task was also reported by Parsons and co-workers and Yamamoto and co-workers [13], [14], [15]. To conclude, however, that men and women are equally interested in infant faces is possibly premature. Hahn and co-workers used a key-press task, in which participants had to respond to cute and less cute infant faces, as well as to attractive and less attractive adult faces. While women exerted the same amount of effort to see cute infant and attractive opposite sex faces, men worked harder to see attractive female faces but showed no effort to look at cute infants, and even worked to not see less cute infant faces. This suggests that the incentive salience of infant faces is more volatile for men then for women and dependent on the context it is presented in.

Overall, for the cross-sectional study, we found no difference between neutral and happy facial expressions in the rating task, but a significant (albeit small) effect for emotion in the key-press task (see Figure 3). It should be noted that the preference of happy faces over neutral faces was not specific to a particular group, ruling out hormonal status as a potential cause for the significant emotion effect. The main finding of the cross-sectional study is the lack of support for the hypothesis that reproductive hormone level modulates the processes of ‘liking’ and ‘wanting’.

There is a caveat. For the cross-sectional study, we tested an unselected group of regularly cycling women from different stages of the menstrual cycle. Given that hormone levels vary widely during the menstrual cycle, there is the possibility that potential differences in hormone-modulated performance between women taking and not taking oral contraceptives are obscured. In a previous study we did find a significant difference in cuteness sensitivity between an unselected group of regularly cycling women and women taking oral contraception [8]. This suggests that comparing unselected groups of regularly cycling women with women taking oral contraceptives is, in principle, valid. The impact of different forms of oral contraceptives on performance merits further investigation.

Nevertheless, to address variation of reproductive hormone levels during the menstrual cycle more systematically, we tested the same women in the menstrual, follicular and luteal phases of their menstrual cycle in a follow-up study. Given that the levels of reproductive hormones peak differentially during the menstrual cycle, with level of oestrogen being relatively high in the follicular phase, level of progesterone being relatively high during the luteal phase, and levels of both hormones being relatively low during the menstrual phase, we would expect changes in ‘liking’ and ‘wanting’ across the menstrual cycle, if ‘liking’ and ‘wanting’ are modulated by reproductive hormones.

Planned non-parametric comparisons of the overall cuteness ratings showed no significant differences between menstrual cycle phases. Effect sizes for this were moderate to low. Looking at happy faces and neutral faces separately, there were no significant differences for happy and neutral faces between cycle phases. We also compared the cuteness ratings for the three levels of cuteness for both the neutral and happy expressions separately. All between-cycle phase comparisons gave non-significant results with small to very small effect sizes. We were also interested in whether happy and neutral faces were rated differently in the follicular, luteal or menstrual phase of the cycle. All three comparisons revealed non-significant differences with very low effect sizes. These findings very much mirror the results from the cross-sectional study.

For the key-press task we found a significant cuteness effect with a high effect size, in that cute infant faces were looked at for longer, but no differences in cuteness ratings between the three stages of the menstrual cycle. This result suggests that there is no major influence of reproductive hormone status on ‘liking’ and ‘wanting’. We found a trend towards significance for the emotion x menstrual cycle phase interaction, in that in the menstrual phase, the happy faces were looked at for a longer time. The effect size of this interaction was small to moderate. Given that reproductive hormone levels are low during the menstrual phase of the cycle, this finding speaks against the suggested link of elevated reproductive hormone level and increased incentive value of infant faces.

In some women the menstrual phase is associated with mood changes, which can range from mild dysphoria (Premenstrual Syndrome - PMS) to clinical depression (Premenstrual Dysphoric Disorder) [17]. It may well be that looking at happy infant faces helps to overcome cycle-related low mood by activating the reward system. This is an interesting perspective for future research, but remains merely speculative at the moment, because we did not assess mood.

In summary, while cuteness, and to a lesser extent happiness, determines the aesthetic and incentive salience of infant faces, we found no evidence of hormonal modulation of ‘liking’ and ‘wanting’. These findings contrast with previous results, which show that sensitivity for small differences in infant cuteness are linked to reproductive hormone status [8], [9]. Future studies have to address the link between hormone-modulated sensitivity for infant cuteness and the aesthetic and incentive value of infant faces using the same participants and the same experimental stimuli.

### Limitations

There are some limitations to the study. In our cross-sectional study we used unselected groups of regularly cycling women and women taking various kinds of oral contraceptives. This might obscure reproductive hormone-modulated performance. We also used a potentially less sensitive 5-point Likert scale, which also may have obscured differences in cuteness ratings between groups. The use of a more sensitive Visual Analogue Scale has to be considered in future studies. Future studies should also use viewing time and key-presses as dependent variables to make comparisons across studies easier.

It should also be noted that we tested participants who were not parents, and that we used static pictures of infants. To draw conclusions about the actual care-giving behaviour of men and women is therefore difficult on the basis of our data and needs more ecologically valid experimental paradigms.

## References

[1] Lorenz K (1943) Die angeborenen Formem möglicher Erfahrung. Z Tierpsychol 5: 233–409.

[2] Karraker K, Stern M (1990) Infant physical attractiveness and facial expression: Effects on adult perceptions. Basic Appl Soc Psych 11: 371–385.

[3] Stephan CW, Langlois JH (1984) Baby beautiful: adult attributions of infant competence as a function of infant attractiveness. Child Dev 55: 576–585. 6723448

[4] Hildebrandt KA, Fitzgerald HE (1978) Adults Responses to Infants Varying in Perceived Cuteness. Behav Processes 3: 159–172. 24924654 10.1016/0376-6357(78)90042-6

[5] Power TG, Hildebrandt KA, Fitzgerald HE (1982) Adults' responses to infants varying in facial expression and perceived attractiveness. Infant Behav Dev 5: 33–44.

[6] Glocker ML, Langleben DD, Ruparel K, Loughead JW, Gur RC, et al (2009) Baby schema in infant faces induces cuteness perception and motivation for caretaking in adults. Ethology 115: 257–263. 22267884 10.1111/j.1439-0310.2008.01603.x PMC3260535

[7] Langlois J, Ritter J, Casey R, Sawin B (1995) Infant attractiveness predicts maternal behaviours and attitudes. Dev Psychol 31: 464–472.

[8] Sprengelmeyer R, Perrett DI, Fagan EC, Cornwell RE, Lobmaier JS, et al (2009) The Cutest Little Baby Face: A Hormonal Link to Sensitivity to Cuteness in Infant Faces. Psychol Sci 20: 149–154. 19175530 10.1111/j.1467-9280.2009.02272.x

[9] Lobmaier JS, Sprengelmeyer R, Wiffen B, Perrett DI (2010) Female and male responses to cuteness, age and emotion in infant faces. Evol Hum Behav 31: 16–21.

[10] Aharon I, Etcoff N, Ariely D, Chabris CF, O'Connor E, et al (2001) Beautiful faces have variable reward value: fMRI and behavioral evidence. Neuron 32: 537–551. 11709163 10.1016/s0896-6273(01)00491-3

[11] Berridge KC, Robinson TE (2003) Parsing reward. Trends Neurosci 26: 507–513. 12948663 10.1016/S0166-2236(03)00233-9

[12] Levy B, Ariely D, Mazar N, Chi W, Lukas S, et al (2008) Gender differences in the motivational processing of facial beauty. Learn Motiv 39: 136–145. 10.1016/j.lmot.2007.09.002 PMC3838871 24282336

[13] Yamamoto R, Ariely D, Chi W, Langleben DD, Elman I (2009) Gender differences in the motivational processing of babies are determined by their facial attractiveness. PLoS ONE 4: e6042. 19554100 10.1371/journal.pone.0006042 PMC2698285

[14] Parsons CE, Young KS, Parsons E, Dean A, Murray L, et al (2011) The effect of cleft lip on adults' responses to faces: cross-species findings. PLoS ONE 6: e25897. 22016785 10.1371/journal.pone.0025897 PMC3189949

[15] Parsons CE, Young KS, Kumari N, Stein A, Kringelbach ML (2011) The motivational salience of infant faces is similar for men and women. PLoS ONE 6: e20632. 21655195 10.1371/journal.pone.0020632 PMC3105111

[16] Hahn AC, Xiao D, Sprengelmeyer R, Perrett DI (2012) Gender differences in the incentive salience of adult and infant faces. Q J Exp Psychol (Hove). 10.1080/17470218.2012.705860 22928658

[17] Halbreich U, Borenstein J, Pearlstein T, Kahn LS (2003) The prevalence, impairment, impact, and burden of premenstrual dysphoric disorder (PMS/PMDD). Psychoneuroendocrinology 28 Suppl 31–23. 10.1016/s0306-4530(03)00098-2 12892987

